# A Logistic Regression Model for Predicting Axillary Lymph Node Metastases in Early Breast Carcinoma Patients

**DOI:** 10.3390/s120709936

**Published:** 2012-07-23

**Authors:** Fei Xie, Houpu Yang, Shu Wang, Bo Zhou, Fuzhong Tong, Deqi Yang, Jiaqing Zhang

**Affiliations:** Breast Disease Center, Peking University, People's Hospital, Beijing 100044, China; E-Mails: dr.xiefei@gmail.com (F.X.); hope2002399@gmail.com (H.Y.); zhoubo@pkuph.edu.cn (B.Z.); tongfuzhong@126.com (F.T.); renminruxian@163.com (D.Y.); ccc1452@sina.com (J.Z.)

**Keywords:** breast cancer, axillary metastases, predictive model, logistic regression, lymph node staging

## Abstract

Nodal staging in breast cancer is a key predictor of prognosis. This paper presents the results of potential clinicopathological predictors of axillary lymph node involvement and develops an efficient prediction model to assist in predicting axillary lymph node metastases. Seventy patients with primary early breast cancer who underwent axillary dissection were evaluated. Univariate and multivariate logistic regression were performed to evaluate the association between clinicopathological factors and lymph node metastatic status. A logistic regression predictive model was built from 50 randomly selected patients; the model was also applied to the remaining 20 patients to assess its validity. Univariate analysis showed a significant relationship between lymph node involvement and absence of nm-23 (*p* = 0.010) and Kiss-1 (*p* = 0.001) expression. Absence of Kiss-1 remained significantly associated with positive axillary node status in the multivariate analysis (*p* = 0.018). Seven clinicopathological factors were involved in the multivariate logistic regression model: menopausal status, tumor size, ER, PR, HER2, nm-23 and Kiss-1. The model was accurate and discriminating, with an area under the receiver operating characteristic curve of 0.702 when applied to the validation group. Moreover, there is a need discover more specific candidate proteins and molecular biology tools to select more variables which should improve predictive accuracy.

## 1. Introduction

Axillary lymph node metastasis is one of the most important prognostic determinants for patients with breast cancer [1]. Axillary lymph node dissection (ALND) had been the standard staging and therapeutic procedure for many years. However, there are significant short- and long-term morbidities after ALND, including arm lymphedema, shoulder range of motion (ROM) impairment, pain, and numbness or paresthesias [2,3]. In recent years, the proportion of node involvement patients has been constantly decreasing as breast cancer is more often diagnosed at an early stage. Surgical ALND procedures have been less relevant in treatment selection because of the introduction of sentinel lymph node biopsy (SLNB) [4], which offers information on the axillary lymph node status with significantly lower morbidity compared to that associated with ALND [5]. However, the morbidity after SLN biopsy is not negligible; lymphedema and impaired shoulder range of motion remain a clinically relevant sequela after SLNB [6,7]. Furthermore, Sentinel lymph node biopsy has an inherent false-negative rate of 5%–10% that cannot be entirely eliminated [8,9]. If any SLN is positive, the standard therapy remains completion ALND. Indeed, many patients with positive SLNs do not show other axillary lymph node metastasis. In approximately 50%–65% of patients, SLN is the sole site of regional nodal metastasis [10,11]. As a matter of fact, women with breast cancer do not benefit from removal of non-involved axillary lymph nodes. According to the ACOSOG-011 data, among patients with limited SLN metastatic breast cancer treated with breast conservation and systemic therapy, SLND compared with ALND did not result in inferior survival (5-year overall survival was 92.5% *vs.* 91.8%; 5-year disease-free survival was 83.9% *vs.* 82.2%) [12]. On the other hand, SLN positive patients will receive systemic therapy regardless of the presence of any additional nodal metastasis; the therapeutic impact of the number of the positive lymph node is minimal [13], so there is also a debate about the necessity for complete ALND in every patient with metastatic SLN.

Management strategies that avoid axillary invasive procedures are needed for lymph node negative patients. If we can predict the state of the axillary lymph nodes before SLNB, individuals who are axillary negative could avoid the unnecessary axillary operation. However, the preoperative clinical and imaging examinations of the axilla are rather poor for predicting axillary lymph node involvement. As we know, lymph node metastasis is a multifactorial event. Within the recent years, a number of studies have investigated the factors and their predictive value for predicting non-sentinel lymph node metastasis (NSLN). The Memorial Sloan Kettering Cancer Center (MSKCC) nomogram showed a receiver-operator characteristic curve (ROC) of 0.76 [14]. Three additional nomograms from France, Tenon Hospital, Paris [15], Cambridge, England [16] and Stanford, USA [17] have been developed more recently. The predictability of these four different nomograms on NSLNM in breast cancer patients with positive sentinel lymph node biopsy was evaluated in a multi-centre study, the AUC values were 0.705, 0.711, 0.730, and 0.582 for the MSKCC, Cambridge, Stanford, and Tenon models respectively [18]. Prediction model (nomogram) in predicting the likelihood of SLN metastasis was also be investigated by using logistic regression. A study consist 4,608 breast cancer procedures of axillary SLN biopsy was performed at MSKCC, a nomogram associated with tumor type, LVI, tumor size, tumor location, age, multifocality, and ER and PR status was developed. The area under the receiver operating characteristic curve was 0.754 [19].

The goal of this study is to characterize the various clinicopathologic features in cases of early breast cancer by using a logistic regression model, in order to identify the factors that might help in predicting the status of the axillary lymph node. Immunohistochemistry technique was used to study hormone receptor (ER, PR), human epidermal growth factor receptor 2 (HER2), tumor metastasis /invasion related genes (Kiss-1, nm-23, Cath-D), oncogenesis related gene (p53), and proliferation related gene (Ki-67). Fluorescence in situ hybridization (FISH) was used for those where IHC staining for HER2 is equivocal or 2+. We also take some important clinical characteristics (e.g., tumor size, age, pathologic tumor grading, menopausal status) into account.

## 2. Materials and Methods

Detailed patient information is described in Table 1. Seventy female breast cancer patients treated in Peking University People's Hospital between December 2006 and July 2007 were enrolled according to the following criteria: primary invasive breast carcinoma, <5 cm in diameter at pathological examination, clinically node negative(clinical breast examination and imageological diagnosis), negative history of previous cancer. All patients were treated with either breast-conserving surgery (n = 42) or modified radical mastectomy (n = 28) including axillary lymph node dissection (at least 10 nodes resected). All patients underwent diagnostic imaging, including mammography, ultrasound and/or Magnetic Resonance Imaging (MRI). None of the patients had received neo-adjuvant chemotherapy.

Tumor tissues were obtained from paraffin embedded specimens. We took age at diagnosis, menopausal status, tumor size, histological grading, lymph node involvement and status of estrogen receptor (ER), progesterone receptor (PR), HER2/neu, Kiss-1, nm-23, p53, ki-67 and Cath-D into account. Among the 70 breast cancer patients, there were 62 infiltrating ductal carcinomas, six infiltrating lobular carcinomas, and two mucinous carcinomas, 44 had lymph-node-negative disease and 26 had lymph-node-positive disease (Table 1). All tissue samples had been routinely fixed in 4% neutral formalin and embedded in paraffin and immunohistochemical (IHC)-stained sections were routinely performed. An IHC score of greater than 3 (at least 10% weakly positive cells) was used to define ER/PR positivity [20]. HER2/neu was scored with the system that has been widely used in clinical testing (0; 1+≥ 10% cells weakly positive; 2 +≥ moderate homogeneous staining; 3 +≥ strong homogeneous staining). For Kiss-1 and nm-23, The percentage of tumor cells showing each staining intensity was estimated to calculate an intensity score ([0 × %weak] + [1 × %mild] + [2 × %strong]) that could range from 0 to 200. A score ≥ 100 was defined as positive staining and a score <100 wasdefined as negative staining. P53 was “negative” if nuclear staining of tumour cells was <10% and “positive” if ≥ 10%. Ki-67 was “negative” if nuclear staining of tumour cells was ≤ 14% and “positive” if > 15%.

Data was subjected to univariate and multivariate logistic regression using SPSS statistical software version 16.0 (SPSS Inc., Chicago, IL, USA). Prediction criterion was a dichotomous variable indicating the pathologic result of the axillary lymph node dissection revealing either no lymph node metastasis or at least one metastatic axillary lymph node. Factors included in the analysis were categorized as shown in Table 1. Fifty patients (model group) were randomly selected to build the model (Table 2), the remaining 20 patients (validation group) were used to assess the validity of it. A formula for predicting lymph node metastases was developed based on the patients in the modeling group, and then validated with the patients in the validation group. In the modeling group, the logistic regression model was constructed using the backward selection procedure in an attempt to discover the predictors of axillary lymph node involvement.

Goodness-of-fit of the model was evaluated by a Hosmer-Lemeshow (HL) test. Receiver operating characteristic (ROC) curves was used to assess the adequacy of the prediction model and determined an optimal cut-off value (A model with a ROC of 0.5 is equal to the toss of a coin. A model with a ROC of 0.7–0.8 is considered good, whereas an ROC of 0.81–0.9 has excellent discrimination). A P-value < 0.05 was regarded as statistically significant.

The standard logistic regression formula is:

(1) Logit(P)=β0+β1X1+βX2+……+βnXn

where Logit(P)=ln[p/(1 − p)], “P” is the estimated probability of lymph node metastasis, “n” is the number of influence factors, “β” is the influence coefficient, “X” is the influence factor, “β_0_” is a constant. X_n_ is a lymph node metastasis promoting factor when β_n_ > 0, conversely, X_n_ is a lymph node metastasis suppressing factor when β_n_ < 0.

## 3. Results and Discussion

The overall frequency of lymph node metastasis was 37.14%. In the univariate analysis, absence of nm-23 (*p* = 0.010) and Kiss-1 (*p* = 0.001) were associated with lymph node metastasis respectively (n = 70).

Fifty patients were randomly selected for the modeling group, the other 20 patients were placed in the validation group. There was no significant difference between the modeling group and the validation group (40% *vs.* 30%; *p* = 0.434). Univariate analysis was performed to evaluate the association between tumor characteristics and axillary status in the modeling group, first. Absence of nm-23 (*p* = 0.022) and Kiss-1 (*p* = 0.002) were related with lymph node metastases (Table 3).

To avoid omitting significant indicators, factors with a significance of *p* < 0.4 in univariate analysis were considered to entry into the multivariate model [21]. Therefore, seven clinicopathological predictors were involved into the multivariate logistic regression model including menopausal status, tumor size, ER, PR, HER2, nm-23 and Kiss-1. Kiss-1 remained significantly associated with positive axillary node status (*p* = 0.018; Table 4). The final logistic regression model for predicting lymph node metastasis as follows:

(2) $$\text{Logit}(\text{P})=-0.474+1.18{\text{X}}_{2}+1.297{\text{X}}_{3}-0.906{\text{X}}_{5}+1.38{\text{X}}_{6}-0.124{\text{X}}_{7}-1.116{\text{X}}_{8}-2.171{\text{X}}_{9}$$

**Figure 1.**
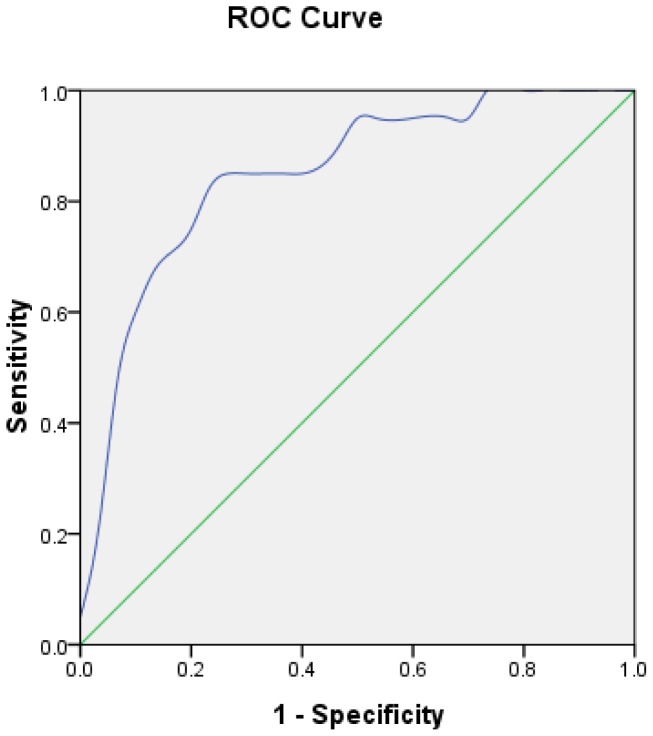
ROC curve calculation for the logistic regression model applied to the modeling group (n = 50).

Goodness-of-fit of the model was assessed by Hosmer-Lemeshow (HL) test. Degree of freedom (df) is 8 (*p* = 0.696), which means the model fitted well. The receiver operating curves (ROC) is shown in Figure 1. The performance of the model was good with an area under curve (AUC) of 0.849. The estimated probability at sensitivity and specificity maximum sum are at a cut-off probability of 0.424, which means if the estimated probability was ≥0.424, a patient was categorised in the lymph node positive group. On the contrary, those with a probability of <0.424 would be classified into the negative group.

**Figure 2.**
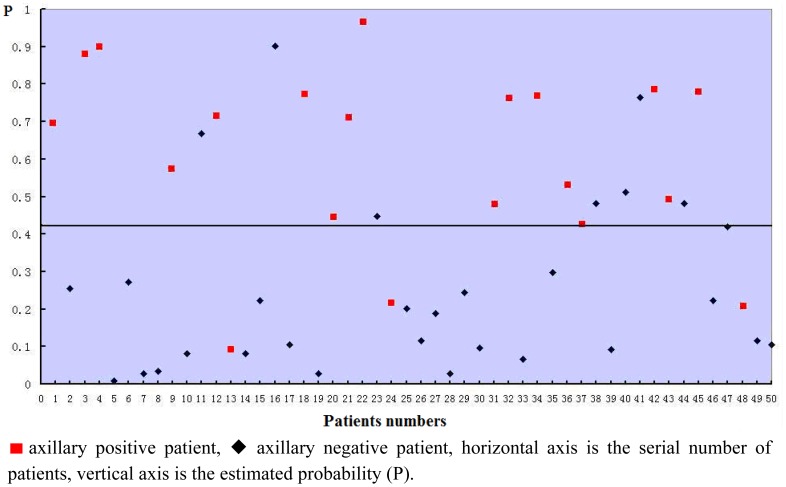
Logistic regression model Scatter diagram (n = 50).

The estimated probabilities of all the 50 patients used for building the model was calculated by the equation and shown in Figure 2. The sensitivity of the model was 85.0%, specificity was 76%, and the accuracy was 80%.

**Figure 3.**
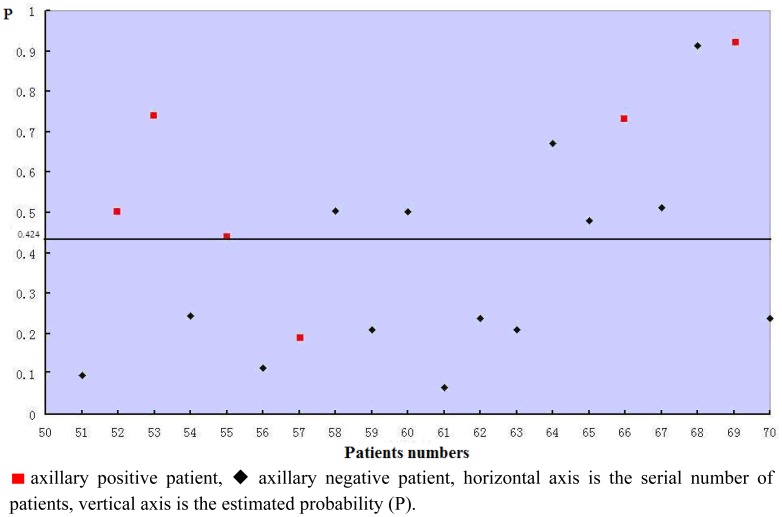
Logistic regression model Scatter diagram (n = 20).

**Figure 4.**
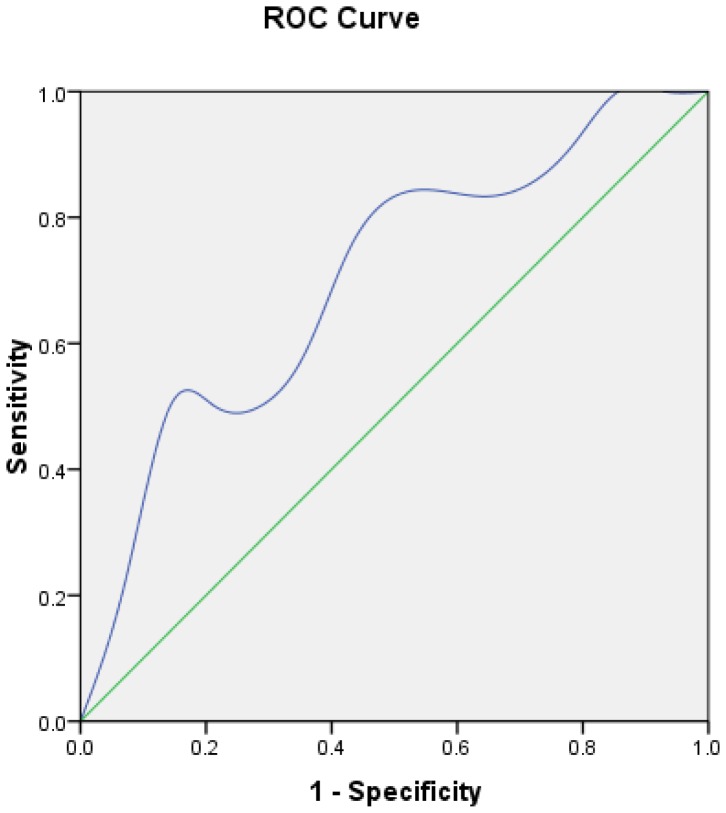
ROC curve calculation for Logistic regression model applied to the validation group (n = 20).

The validity of the logistic regression model was assessed in the remaining 20 patients. Every patient's estimated probability was calculated by the formula and shown in Figure 3. Five of six axillary positive patients' estimated probabilities were >0.424, so the sensitivity of the model was 83.3%. The specificity was 57.14%. The predictive accuracy of the model was 65%. The ROC is shown in Figure 4. The performance of the model was good with an area under curve (AUC) of 0.702.

Nodal staging in breast cancer is a key predictor of prognosis [1]. In the past, the only way to evaluate the lymph node metastases was to perform complete ALND, but at the expense of several functional consequences. SLNB has been shown to be a good procedure to evaluate the axillary lymph node status [22–24]. However, the morbidity after SLN biopsy is not negligible. Furthermore, if any SLN is positive, patients still need to receive ALND as a standard treatment. Moreover, technical failure occurs in about 2%–6% cases necessitating a complete ALND [25,26]. In fact, SLNs is positive only in about 23% of cases [27], the SLNs have been found as the only foci of axillary metastases in about 50%–65% of the patients [10,11]. Consider the high accuracy rate of SLNB, SLNB could be omitted for those negative patients. Also, a portion of SLN positive patients have received unnecessary ALND.

In the past decade, some studies were conducted for the development of nomograms to identify patients with sufficiently low risk of NSLNM (non sentinel node metastasis) who can then avoid ALND. Van Zee *et al.* [14] from the Memorial Sloan Kettering Cancer Center (MSKCC) created a nomogram assessing potential independent predictors of non-sentinel lymph node metastasis (NSLN), which showed an AUC of 0.76. Logistic regression models are at best discussion on prediction of lymph node metastases. A nomogram to predict the probability of having four or more nodes based on patients' pathologic data was developed from the multivariate logistic regression model by the Massachusetts General Hospital group [17]. The study showed having four or more positive lymph nodes was associated with tumor histology, primary tumor size, lymphovascular space invasion, extra-nodal extension, number of involved SLNs, number of uninvolved SLNs, and the size of the largest SLN metastasis. Another prediction model (nomogram) based on a large data set to assist in predicting the presence of SLN metastasis was developed [19]. The model was applied to 1,545 SLN biopsies with an area under the receiver operating characteristic curve of 0.754. Age, tumor size, special type, tumor location, multifocal, nuclear grade, ER and PR were included in the nomogram. However, there is a need discover more specific candidate proteins and molecular biology tools to select more variables which should improve predictive accuracy.

In our study, we found that postmenopausal women, large tumor (>2 cm) and PR positive women appear to have a trend of high risk of lymph node involvement. However, there was no statistical significance. On the contrary, there is a trend of negative correlation between women with positive expression of ER, HER2, nm-23, Kiss-1 and lymph node involvement. Furthermore, nm-23 and Kiss-1 have a significant negative correlation with ALN involvement in the univariate analysis, and absence of Kiss-1 remains significantly associated with positive axillary node status in the multivariate analysis.

Nm-23 protein was originally identified as a metastasis suppressor protein [28]. Nm23 gene located on the chromosome 17 q21.3 (A subunit by nm23-H1 and B subunit by nm23-H2) and encodes nucleoside diphosphate kinase. It has been reported that differential regulation of nm23 by P53 in different cell types is an important component in the molecular mechanisms of tumor metastasis [29,30]. Many previous studies showed low expression of nm-23 is predictive of distant metastasis and appears to be a risk factor of positive axillary nodes [31–33].

Kiss-1 has been identified as a putative human metastasis suppressor gene in melanomas [34]. It has also been suggested as a potential metastasis suppressor in breast cancer cells without affecting tumourigenicity [35]. Mitchell and colleagues [36] proved that the loss of Kiss-1 gene expression in highly metastatic breast cancer cell lines. Kostadima *et al.* [37] also have reported that the positive rate of Kiss-1 is only 3% in node-positive breast cancer, supporting the anti-metastatic role of the Kiss-1. In our study, Kiss-1 is the most important and independent impact factor for lymph node metastases.

Tumor size is an important factor influencing the lymph node involvement in breast cancer. This fact has been confirmed in some studies. Wada *et al.* [38] have reported that tumor size lager than 2 cm is a predictor for tumor involvement in remaining axillary lymph nodes of breast cancer patients with positive sentinel lymph node. An 893 cases analysis also showed that the rate of lymph node metastases increased from 11% to 36% when the tumor size increases from 10 mm to 25 mm [39]. In a series of 157 cases published by Chu *et al.* [40], the rate of non-SLN involvement increased from 13% to 38% from stage T1b to stage T2 tumors. Although there is no statistical differences between tumor size and lymph node involvement in our univariate analysis, the positive lymph node proportion trends to be higher in patients with T > 2 cm than those T < 2 cm (48.3% *vs.* 28.6%), the probable reason for this may be the small sample size of our model, while in multivariate model, the influence coefficient of tumor size is 1.297, the effect weight is just a little bit lower than Kiss-1 and PR.

ER status is both prognostic and predictive factor for breast cancer [41], the presence of ER in a breast tumor is believed to coincide with low lymph node involvement risk [42,43]. According to the SEER report, the proportion of patients ER expression is about 65%–70% [44]. However, in our study, the rate of ER positive cases was only 45.7%, different criteria of ER positive might account for it. According to the Allred scoring of ER status, an IHC score of greater than 2 (corresponding to as few as 1% to 10% weakly positive cells) was used to define ER positivity [20], because of the significant superiority in response to adjuvant endocrine therapy. In our study, consider of the influencing efficacy of ER, IHC score of greater than 3 (at least 10% weakly positive cells) was used as the ER positive critical value.

PR may play a contrasting role from the regression model (OR = 3.975). This observation seems counterintuitive, but it actually is in agreement with the findings of other large studies. Ravdin *et al.* [45] studied data from 26,683 patients and found PR concentrations to be positively associated with risk of lymph node metastasis in their multivariate model. Gann *et al.* [46] analyzed data from 18,025 patients and Viale *et al.* [47] studied the prediction of sentinel lymph node metastases for 4,351 patients, both identified the same conclusion. Actually, human PR proteins exist as two isoforms: termed PR-A and PR-B. The two PR isoforms differ in structure and function [48]. Only a few studies have separately examined PR-A and PR-B expression. Torsten *et al.* [49] have reported that high PR-A: PR-B ratios were 2.76 times more likely to relapse than patients with lower ratios. That might be an explanation for different role of PR in breast cancer lymph node metastasis in different studies.

One could argue that HER2 seems as a metastases inhibitor in our study. The frequencies of lymph node metastases in HER2-positive tumors were lower than those for HER2-negative tumors (31.2% *vs.* 44.1%), but there was no significance (*p* > 0.05). Indeed, HER2 had a very weak effect weight in the regression model (β = 0.124). Also, HER2 was not indispensable for lymph node metastases prediction in the past research [14,17,19].

Some studies have shown that elder or postmenopausal women have a lower risk of ALN involvement [42,43], but our study showed postmenopausal women tend to have high axillary metastases risk. Maybe breast lump in elder women is more often ignored, so it has a long course of diseases before diagnosed.

## 4. Conclusions

The predictive model presented here relies on readily available clinicopathological factors, indicating that some clinicopathologic factors might be used to select patients who were more likely to have positive axillary lymph nodes. However, this model should be applied prospectively to a large number of patients and including additional parameters into the prediction strategy to verify its validity. Maybe in the future, a substantial proportion of women with invasive breast cancer could avoid the morbidity of axillary dissection.

## Figures and Tables

**Table 1.** Patients and tumor characteristics (n = 70).

**Variable**	**Characteristics**		**Code**	**N (%)**
X_1_	Age(year)	≤50	0	28 (40.0%)
		>50	1	42 (60.0%)

X_2_	menopausal status	Premenopausal	0	25 (35.7%)
		postmenopausal	1	45 (64.3%)

X_3_	tumor size(cm)	≤2	0	28 (40.0%)
		>2	1	42 (60.0%)

X_4_	histological grading	I	0	16 (22.9%)
		II-III	1	54 (77.1%)

X_5_	ER	(−)	0	38 (54.3%)
		(+)	1	32 (45.7%)

X_6_	PR	(−)	0	20 (28.6%)
		(+)	1	50 (71.4%)

X_7_	HER2	(−)	0	50 (71.4%)
		(+)	1	20 (28.6%)

X_8_	nm-23	(−)	0	22 (31.4%)
		(+)	1	48 (68.6%)

X_9_	Kiss-1	(−)	0	26 (37.1%)
		(+)	1	44 (62.9%)

X_10_	P53	(−)	0	34 (48.6%)
		(+)	1	36 (51.4%)

X_11_	Ki-67	(−)	0	25 (35.7%)
		(+)	1	45 (64.3%)

X_12_	Cath-D	(−)	0	41 (58.6%)
		(+)	1	29 (41.4%)

**Table 2.** Modeling group patients and tumor characteristics (n = 50).

**Variable**	**Characteristics**		**Code**	**N (%)**	
X_1_	Age (years)	≤50	0	18 (36.0%)
		>50	1	32 (64.0%)

X_2_	Menopausal status	Premenopausal	0	16 (32.0%)
		postmenopausal	1	34 (68.0%)

X_3_	Tumor size (cm)	≤2	0	21 (42.0%)
		>2	1	29 (58.0%)

X_4_	Histological grading	I	0	12 (24.0%)
		II-III	1	38 (76.0%)

X_5_	ER	(−)	0	27 (54.0%)
		(+)	1	23 (46.0%)

X_6_	PR	(−)	0	14 (28.0%)
		(+)	1	36 (72.0%)

X_7_	HER2	(−)	0	34 (68.0%)
		(+)	1	16 (32.0%)

X_8_	nm-23	(−)	0	18 (36.0%)
		(+)	1	32 (64.0%)

X_9_	Kiss-1	(−)	0	15 (30.0%)
		(+)	1	35 (70.0%)

X_10_	P53	(−)	0	25 (50.0%)
		(+)	1	25 (50.0%)

X_11_	Ki-67	(−)	0	17 (34.0%)
		(+)	1	33 (66.0%)

X_12_	Cath-D	(−)	0	30 (60.0%)
		(+)	1	20 (40.0%)

**Table 3.** Univariate analysis of tumor characteristics and lymph nodes involvement (n = 50).

**Variable**	**Characteristics**		**Axillary Nodes**		***x^2^***	***P***

			**Negative Positive**			
X_1_	Age (years)					
		≤50	10	8	0.231	0.630
		>50	20	12		
X_2_	Menopausal status					
		Premenopausal	11	5	0.751	0.386
		Postmenopausal	19	15		
X_3_	Tumor size (cm)					
		≤2	15	6	1.970	0.160
		>2	15	14		
X_4_	Histological grading					
		I	7	5	0.018	0.892
		II-III	23	15		
X_5_	ER					
		(−)	14	13	1.624	0.203
		(+)	16	7		
X_6_	PR					
		(−)	10	4	1.058	0.304
		(+)	20	16		
X_7_	HER2					
		(−)	19	15	0.751	0.386
		(+)	11	5		
X_8_	nm-23					
		(−)	7	11	5.223	0.022
		(+)	23	9		
X_9_	Kiss-1					
		(−)	4	11	9.921	0.002
		(+)	26	9		
X_10_	P53					
		(−)	14	11	0.333	0.564
		(+)	16	9		
X_11_	Ki-67					
		(−)	10	7	0.015	0.903
		(+)	20	13		
X_12_	Cath-D					
		(−)	18	12	0.000	1.000
		(+)	12	8		

**Table 4.** Multivariate analysis of clinicopathological data and nodes involvement (n = 50).

**Variable**	**Characteristics**	***β***	***S.E***	***Wald***	***P***	***OR***	**95.0% *C.I.* for *OR***	

							***Lower***	***Upper***
X_2_	menopausal status	1.182	0.879	1.808	0.179	3.262	0.582	18.282
X_3_	Tumor size	1.297	0.816	2.526	0.112	3.658	0.739	18.108
X_5_	ER	−0.906	0.783	1.340	0.247	0.404	0.087	1.874
X_6_	PR	1.380	0.963	2.052	0.152	3.975	0.601	26.269
X_7_	HER2	−0.124	0.829	0.022	0.881	0.883	0.174	4.488
X_8_	nm-23	−1.166	0.836	1.948	0.163	0.312	0.061	1.602
X_9_	Kiss-1	−2.171	0.921	5.559	0.018	0.114	0.019	0.693
	Constant	−0.474						
